# Extracting Value from Industrial Alarms and Events: A Data-Driven Approach Based on Exploratory Data Analysis

**DOI:** 10.3390/s19122772

**Published:** 2019-06-20

**Authors:** Aguinaldo Bezerra, Ivanovitch Silva, Luiz Affonso Guedes, Diego Silva, Gustavo Leitão, Kaku Saito

**Affiliations:** 1 Postgraduate Program in Electrical and Computer Engineering, Federal University of Rio Grande do Norte, Natal 59078-970, Rio Grande do Norte, Brazil; affonso@dca.ufrn.br; 2 Digital Metropolis Institute, Federal University of Rio Grande do Norte, Natal 59078-970, Rio Grande do Norte, Brazil; ivan@imd.ufrn.br (I.S.); gustavo.leitao@imd.ufrn.br (G.L.); 3 School of Sciences and Technology, Federal University of Rio Grande do Norte, Natal 59078-970, Rio Grande do Norte, Brazil; diego@ect.ufrn.br; 4 Petróleo Brasileiro S.A., Rio de Janeiro 21941-915, Brazil; kaku@petrobras.com.br

**Keywords:** alarm and event management, data science, exploratory data analysis, industry 4.0, monitoring

## Abstract

Alarm and event logs are an immense but latent source of knowledge commonly undervalued in industry. Though, the current massive data-exchange, high efficiency and strong competitiveness landscape, boosted by Industry 4.0 and IIoT (Industrial Internet of Things) paradigms, does not accommodate such a data misuse and demands more incisive approaches when analyzing industrial data. Advances in Data Science and Big Data (or more precisely, Industrial Big Data) have been enabling novel approaches in data analysis which can be great allies in extracting hitherto hidden information from plant operation data. Coping with that, this work proposes the use of Exploratory Data Analysis (EDA) as a promising data-driven approach to pave industrial alarm and event analysis. This approach proved to be fully able to increase industrial perception by extracting insights and valuable information from real-world industrial data without making prior assumptions.

## 1. Introduction

Industry carries the stigma of being a data rich and knowledge poor environment, partly because the sector is inherently conservative about its processes and methods. Studies state that while manufacturing industry generates more data than any other sector of the economy, yet much of it is not harnessed by companies [1,2]. One of them reports an example of one oil-and-gas company which discards 99% of its data before decision makers have a chance to use it. This was substantially underscored by the advent of Smart Manufacturing and its integrated and performance-oriented enterprise with intensive and pervasive application of networked information-based technologies throughout the manufacturing and supply chain [3]. However, smart factories focus mostly on control-centric optimization and intelligence, when a greater intelligence can be achieved by interacting with different surrounding systems that have a direct impact to machinery performance [4].

Current competitive business environment has been forcing industries to process data into timely and valuable information. Recent advances in Data Science and Big Data domains have assisted enterprises in this endeavor of transforming manufacturing value chain by enabling the acquisition of valuable information from raw and massive data originated in industrial plants, supporting operators, analysts and managers in decision making, action planning and continuous improvement of plant operations.

Nowadays, two dominant and interoperable trends drives factory automation: Industry 4.0 and IIoT (Industrial Internet of Things). The former, also known as the 4th industrial revolution, portrays that business objectives, intelligent algorithms, analytics, predictive technologies and cyber-physical systems are teaming together to realize a new thinking of production management and factory transformation [4]. It ultimately represents a convergence of information technology and operational technology in which supply and production chains will dynamically adjust themselves to provide on-demand customization of manufacturing [5]. The latter refers to the adoption of the IT (Information Technology) domain IoT (Internet of Things) paradigm in manufacturing by joining sensors and actuators, control systems, communication facilities, machine-to-machine, data analytics and security mechanisms together through embedded technology [6]. By promoting the holistic integration of intelligent equipment, intelligent systems and intelligent decision-making, a network of machines, materials, workers, and systems is settled to ultimately achieve smart manufacturing in the context of Industry 4.0 [7].

Much related with those above-mentioned trends, also emerges the broader and abstract concept of Internet of Everything (IoE), in which people, process and data rather than only things are brought together to make networked connections more relevant and valuable in order to reach more sustainable ecosystems [8,9]. In that respect, technologies and applications related to Data Science and Big Data domains have been playing a crucial role [6,10].

The complexity of IT domain technologies, infrastructures and programming models related to those above mentioned trends may hinder the adoption of them in the industrial domain [11]. Much remains to be achieved in fitting the already well-established IT domain Big Data paradigm to industrial reality and needs. This consists basically in elucidating what to do with industrial data and how to do it, once the industry domain differs in several aspects from the other fields where the IT domain Big Data approach has typically and solidly been employed, such as social media, socioeconomics, marketing, e-government and e-commerce, health, economy and politics, to name a few. In this perspective, recent literature have come on the subject of Industrial Big Data with a particular regard to problems and challenges to be overcome in face of the indefeasible absorption of this new paradigm in automation. Proposals of suitable Big Data approaches and architectures to meet industrial needs [4,12,13,14,15] suggests Industrial Big Data is still a developing field of study.

A fact is that the constant growth, technological advances and paradigm shifts, innate to industry, has pushing the sector towards the undeniable reality of data orientation. Coping with that, this work proposes a data-driven approach in line with those above-mentioned trends, to be applied to a specific class of industrial data: event and alarm logs from alarm management systems. Although this kind of data comes in great magnitude and relates to important industrial plant entities, it is normally overlooked in operational processes. In the case of this study, data is composed mainly of categorical and textual entries generated in a crude oil processing plant. The proposed approach is mainly founded on Exploratory Data Analysis (EDA), an elementary technique founded on making data speak for itself, and makes use of the powerful and versatile data analysis ecosystem currently provided by Python and R languages (endowed with a thriving portfolio of libraries) to enrich EDA with adequate and convenient graphical ways of reviewing its results.

The remainder of the paper is structured as follows. Section 2 provides a brief approach to the concepts that support the understanding of this work. Section 3 proposes the use of EDA as a primer tool in alarm and event analysis and brings together some potential EDA approaches applied to a real world data set. Section 4 raises some discussion about the results and finally Section 5 concludes the paper and indicates future works.

## 2. Paper Background

The scientific area, the source domain of target data and the chosen approach are basic topics to be clarified in context of this research.

### 2.1. Data Science and Big Data in Industry

In the wide sense, Data Science refers to the interdisciplinary set of techniques, technologies, processes employed to extract insights and knowledge from data, assisting the decision-making process [16]. Supporting the development of Data Science practices emerges the concept of Big Data, which refers basally, to the scientific domain of computation applied to large, complex and diverse data sets [17]. Although both concepts are commonly fused or synonymized in literature, they are in fact related and complementary to each other. As Big Data is meant to scale Data Science capabilities, they can be seen as cores of current data-driven analysis approaches. While Data Science gather the broad areas of algebra, statistics, engineering and computer science as basal tools in data analysis processes, Big Data stands on the means of making this data analysis computationally viable in the current multiple-sourced data deluge scenario.

As traditional “small data” computational approaches are proven to be ineffective in dealing with a high magnitude data volume [18], new coping approaches and a differentiated computational infrastructures are demanded. This refers basically to the ability of properly handling capture, storage, management, querying, share and visualization of this huge amount of data. This aptitude is made possible through the advent of new computing paradigms such as cloud computing, jungle computing and fog computing, which are elementally based on distributed, parallel and high performance computing [19].

At this point, it is important to enlighten that data-driven approaches have been supporting decision-making processes over the last years in a myriad of sectors and this was enabled by technologies pioneered in the IT realm [12]. Not unlike, data-driveness has also made its path through industry. It happened in a natural way, once manufacturing generates and stores more data than any other sector [12] and given the not so new industry convergence to IT-bond technologies. Recently, with the emergence of Industry 4.0 and IIoT as paradigm shifts in manufacturing, together with the resulting increase in data magnitude and importance, Data Science and Big Data have gained leading roles.

In that regard, Reis and Gins [20] present a comprehensive retrospective work on the trends and paradigms that have headed industrial process monitoring over the last century culminating in the emergence Industry 4.0 and Big Data as enablers for a performance boost in operational, economic, market-related, safety and environmental aspects. Colombo et al. [21] state that the Big Data empowering of cyber-physical systems allow massive amounts of data to be acquired and analyzed for the finest details of processes as well as Data Science approaches on the available Big Data are expected to have a wide impact on the way cyber-physical systems are designed and operated. These approaches can be teamed to provide new insights for the industrial processes, leading to improvements in enterprise operations and identification of optimization opportunities.

As discussed in [12], current industrial computing infrastructure, in terms of management and processing of plant data, is mainly focused on collecting, selecting and storing data at appropriate rates, preserving historical series in on-demand access repositories. Mostly due to a design restriction, any additional processing such as deeper queries or analysis are beyond the capacity of typically installed computing infrastructure. Thus, a current trend in industrial systems refers to the use of different Big Data precepts as a mean to process this huge amount of data already generated in industrial plants which cannot be processed with a conventional infrastructure. This paradigm shift is necessary to put industry on track of the crescent data inundation scenario.

Industrial Big Data is an already well known concept which refers essentially to the absorption of Big Data in Industry. As surveyed in [13], it inherits the defining characteristics of general purpose Big Data concept such as volume, variety, velocity, variability and veracity (5 Vs), as well as extends this concept by adding new Vs: visibility, which regards to the discovery of unexpected insights of existing processed data; and value, which puts emphasis on creating new value from massive data. In that work is also stated that industrial data is more structured, correlated and ready to analysis since it is generated by automated equipment in more controlled environments and processes.

Among several masses of plant-generated data which share these Big Data aspects in modern industry environment are alarm and event logs, a potential a source of knowledge whose exploitation is the main objective of this work.

### 2.2. Industrial Event and Alarm Data

Although being a sector that traditionally produces more data than any other, manufacturing has been experiencing an exponential growth of plant-related data and information production, mainly due to the technological evolution of automation systems [22]. Jargons from IIoT and Industry 4.0 domains such as “data is everywhere”", “instrumentation everywhere” and “connecting everything” are premises which corroborate the fact that modern industries have to deal with a massive (and crescent) amount of data.

When it comes to the mass of data generated in automation, the image of a large and diverse repository of process variables data from field devices (sensors and actuators) comes to mind. Nevertheless, another kind of plant generated data also comes in a considerable amount: alarm and event logs. As these logs reflect time-lined streams of all relevant plant episodes regarding a myriad of plant elements, they may carry important information that should not be took aside.

**Figure 1:**
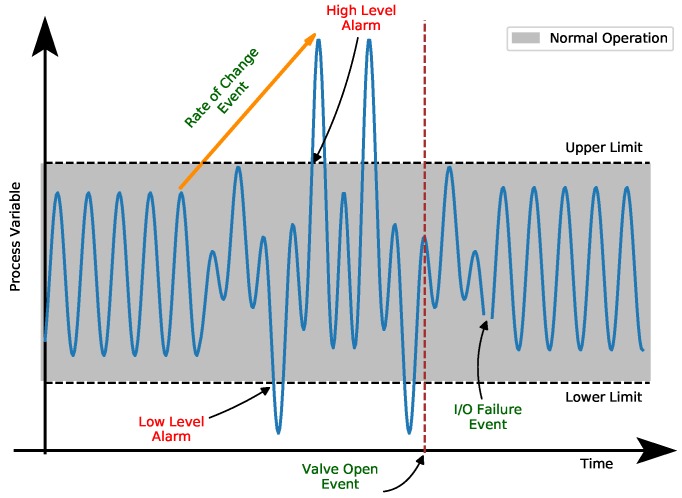
Alarms and events in the context of a monitored process variable.

In industry, an event, without loss of generality, consists of any relevant occurrence within the operational scope of a monitored system. Events evince general plant operation circumstances and normally do not require explicit acknowledge or intervening actions. Alarms, in turn, are audible or visible means of indicating equipment malfunction, deviations in the process or abnormal conditions, requiring a response from operators [23]. Thus, alarms stress out problems and consequently demand series of preventive or corrective actions from operators while events represent any detectable occurrences or changes in the system which may or not be bound or associated to alarms. From process safety formalism, alarms are fundamental barriers in risk control systems designed to detect process parameter deviations [24]. Figure 1 helps to clarify the above mentioned conceptual differences between alarms and events in the context of a fictitious monitored process variable.

Despite being a well established matter in automation, alarm management is a mostly undervalued and misused aspect whose philosophy industry has been very conservative about [25]. In a modernizing contribution to the area, ANSI/ISA-18.2 standard [23] proposes regulations on series of alarm management aspects and addresses old problems regarding definitions, classifications, requirements, life cycles, activities and work processes as well as settles divergences from existent but not so specific regulations in the broad area. In particular, ANSI/ISA-18.2 advises a change in alarm management philosophy by tethering the area to the evolution of work processes instead of focusing only on hardware or software issues. One of the proposals refers to the adoption of advanced analysis methods in monitoring, evaluation, audit and benchmark stages of the life cycle of alarm management systems.

As both events and alarms are meant to verbalize all relevant plant episodes, their related registries constitute a gigantic mass of data which is usually not properly processed. Although these records are closely related to plant operation monitoring, only few of them (mostly alarms) are brought to the attention of operators and analysts through Human-Machine Interfaces (HMIs) of Supervisory Control and Data Acquisition (SCADA) systems and Distributed Control Systems (DCS). Even so, in some incident scenarios, operators may experience an unmanageable data avalanche from alarm management systems which can therefore lead to inadequate operational actions or decisions [26]. The ineffectiveness of alarm management systems in revealing problems is also pointed as possible causes of various accidents in industry [22,27].

It is clear, therefore, that analysis should go beyond the aggregation and presentation of bare alarm and event data in HMIs from traditional alarm management systems, since important information may be hidden from the HMIs scope. In the same way, visual or query-based inspection of raw data is normally error prone due to registry large amount and the tabular format which is poorly suited to human cognition. Furthermore, much attention has been devoted to optimal parameterization and configuration of alarm systems in order to make process operations more efficient and safe [28] while plant events, although often more numerous, do not receive the same attention. Although dealing with industrial events is a common matter in alarm management specialized literature, event data is commonly fated to be stored in loggers or historians and taken into account only when further analysis is needed to elucidate a plant incident or deviation pointed out by an alarm [25,26,28]. Being an important source of information, event data could be more proactively exploited to improve operational performance and governance of an organization. Corroborating with that, specialized literature on process safety and asset integrity advocates that alarm and event data can be used to implement some lagging and leading Key Performance Indicators (KPIs), which are performance metrics that provide evidences of a company’s performance in managing its key risks [24,29].

Thus, a more purposive use of alarm and event related data, under the aegis of current practices in Data Science, may yield an invaluable value from a commonly overlooked data mass. Among this practices, Exploratory Data Analysis (EDA) stands out as an essential methodology for relevant information disclosure, especially in early analysis stages.

### 2.3. Exploratory Data Analysis (EDA)

**Figure 2:**
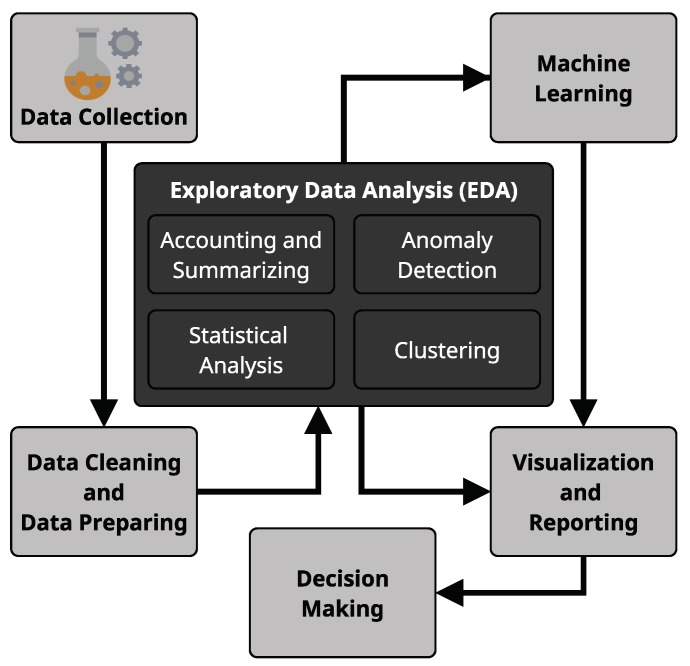
Typical Data Science workflow.

EDA plays an important role in the process of data analysis. It is a key initial step in both explanatory and predictive modeling as it consists on summarizing data numerically and graphically and consequently preparing data for the more formal modeling steps [30]. By summarizing and accounting data, EDA can promptly deliver useful information, find patterns and uncover general relationships which may guide further analysis and potentialize its results. Figure 2 illustrates the role of EDA in a typical Data Science workflow.

After data formatting and preparation basic steps, the classic and strictly data-driven EDA approach makes use of data investigation techniques in the search for interesting information and relationships, from an actively incisive approach, with a real emphasis on the discovery of the unexpected [31]. EDA isolates data patterns and characteristics and reveals them vigorously to the analyst [31], without needing prior knowledge or pre-specified hypotheses, in other words, without requiring anticipated and well defined questions to be directed to the data [32]. That is, EDA cannot lead to definitive conclusions but is an essential first step in understanding data [33].

Basic EDA methods try to make the data more easily and effectively manageable by the user, whether statistician or non-statistician [34]. Hence, a key component in EDA is the employment of various graphical methods to conveniently present data analysis results, combining the sharp human graphic perception with available computational power and versatility. In practice, in order to accomplish its objectives, EDA associates quantitative and qualitative methods from classical statistic approach with graphical analysis [35]. EDA can be compared to a detective work: it is the process of gathering evidence as a hypothesis generation step that precedes the step of Confirmatory Data Analysis (CDA), which is comparable to a court trial and focus in evidence evaluation using traditional statistical tools such as significance, inference, and confidence (hypothesis testing) [31].

EDA is a broad area with a large variety of approaches, methodologies and techniques which can be straightforwardly implemented nowadays due to the great availability of open-source tools and libraries for data analysis. An work on systematizing a few of these approaches is made explicit in the next Section to attest potential of EDA as an ally in obtaining an insightful panoramic view of study data, with focus on their visual demonstrations.

## 3. Alarm and Event Data Analysis

Bearing in mind EDA principles, data was submitted to a groping process in which low-level data, typically too voluminous and confusing to be perceived by humans, is mapped into other more compact and understandable formats in order to ensure a better understanding of data. Data exploration was carried out to pursue the comprehension of the underlying structure of data mainly by assessing quantitative and qualitative aspects as well as depicting variables relationships. Analysis is hence structured in very basic pipeline composed of three stages: data selection, data preparation and visual analysis of data.

### 3.1. Data Selection

In this study, target data is a representative database of event and alarm logs coming from of a petrochemical plant supplied by field specialists. The data set under analysis consists of 1,020,765 records (rows) of 16 variables (columns) concerning events and alarms in a proportion of approximately 40% and 60%, respectively, in the context of a scheduling horizon for an important operational scenario occurred within a interval of approximately 3 days. These entries refer to time-stamped observations of categorical variables for relevant plant entities such as sensors, actuators and controllers. The variables refer to attributes of the automation assets, mainly related to categorizations, hierarchies, states and descriptions. Table 1 shows some sample entries of the data set in tabular format. In this table, some columns were omitted for the sake of limited horizontal space and data is sanitized to hide sensitive information.

### 3.2. Data Preparation

Real-world data is collected, structured and stored with convenience to the domain rules and restrictions in which it is generated. However, data-gathering methods are often loosely controlled, yielding raw and messy data from a practical data analysis perspective. Typically, a series of pre-processing steps are needed to prepare data for further analysis stages. Not unlike, despite being well structured, target data has some quality issues that were addressed in this stage.

#### 3.2.1. Pre-Processing and Transformation

The pre-processing step included basic operations of data cleaning and data editing. With the superficial data knowledge acquired in initial steps of data appreciation and manipulation, it was possible to formulate a set of edit rules for a computer-assisted regularization of corrupted, inadmissible and wrongly encoded records. Accounting and properly formatting of time information was also performed in this stage to favour upcoming time-based analysis. A final step in this stage comprised the set up of new aggregation and generalization helper attributes derived from original data to improve data set quality and ease analysis. For instance, a new categorical helper higher level attribute related to the part of the day was created from time stamps and a new numeric supplementary attribute was calculated to register the uniqueness of a record.

#### 3.2.2. Missing Data Analysis

Data analysis is as good as the data it depends on. So, an important and primal stage in EDA is data quality analysis whose key component is missing data investigation. Missing data can be defined as data values which are not present for a variable in an observation. Missing data can come from a variety of reasons such as equipment failures, configuration errors, communication problems and even human error. By accounting and considering these data absences in analysis, one can create a situational awareness of overall data quality, which can be informative and help to improve data analysis. Knowing the missing data landscape and patterns of a data set can also guide missing data handling strategy in next analysis steps.

**Figure 3:**
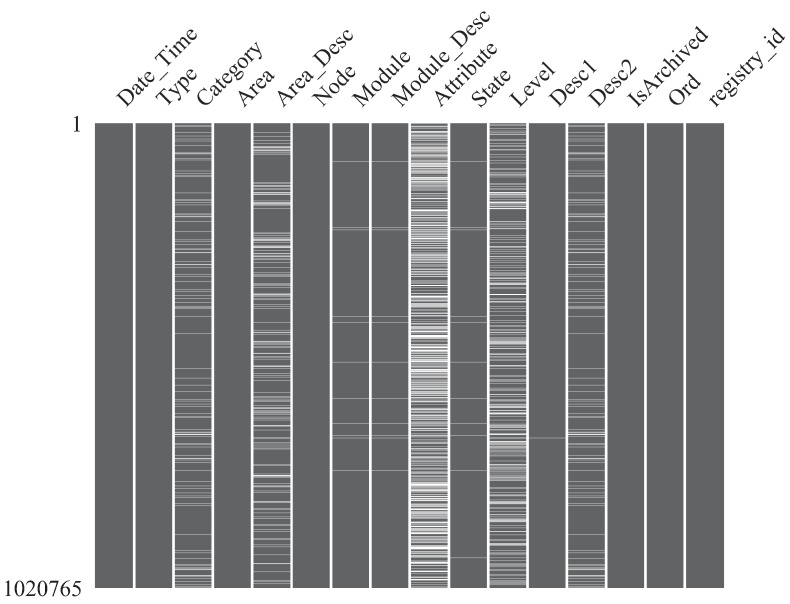
Nullity matrix showing missing data dispersion over variables.

Python package *missingno* [36] is meant for understand missing data through a variety of tools committed to data set completeness visualization. In order to obtain an overview of the missing data on target data set, the nullity matrix shown in Figure 3 was built. It yields a macro view of missing data patterns and dispersion (white lines) over data and shows that data absences are not highly row-wise or column-wise concentrated.

**Figure 4:**
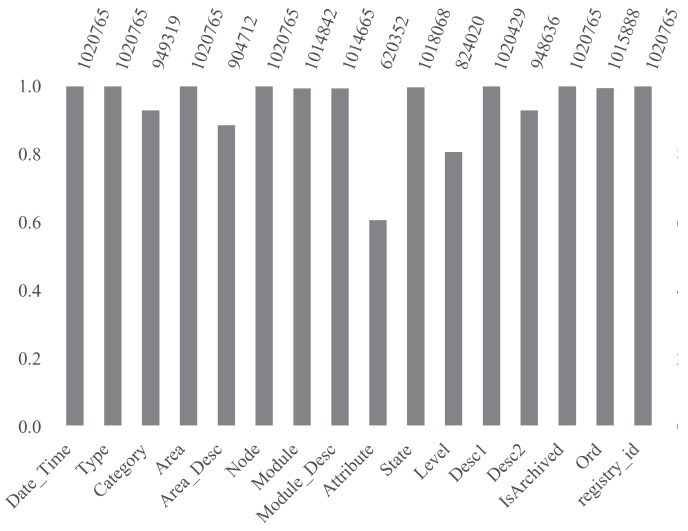
Bar plot showing missing data account and proportions.

Missing data accounting and proportions for each variable can be demonstrated by the enhanced bar chart show in Figure 4. It is possible to notice, for instance, that columns *Attribute* and *Level* have a considerable absent data percentage of about 40% and 20%, respectively.

**Figure 5:**
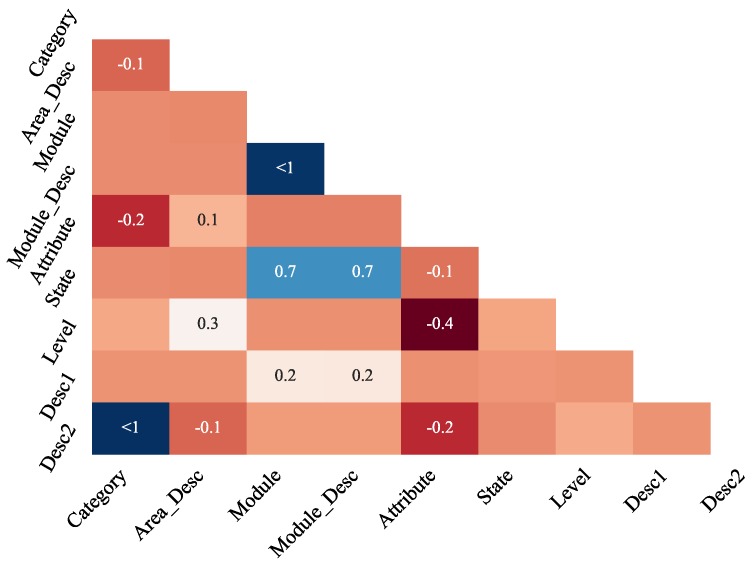
Nullity correlation heatmap.

Going deeper into missing data analysis, nullity correlations can be disclosed by a correlogram as depicted in Figure 5. Also built with *missingno* package, this correlogram aims to graphically evidence how strongly the presence or absence of one variable affects the presence or absence of another.

In that map, nullity correlation (using Pearson’s method) ranges from −1 to 1, where values closest to −1 represent a negative correlation (if for one variable data appears, for the other data is more likely to be missing) and values closest to 1 represent a positive correlation (if for one variable data appears, for the other data is more likely to also appear), whereas 0 represents no nullity correlation (not printed in the map). Variables with no missing data or always empty are omitted in the graph. From Figure 5 it is possible to notice, for example, that columns *Module* and *Module_Desc* have a strong positive nullity correlation while *Level* and *Attribute* have a moderate negative correlation.

**Figure 6:**
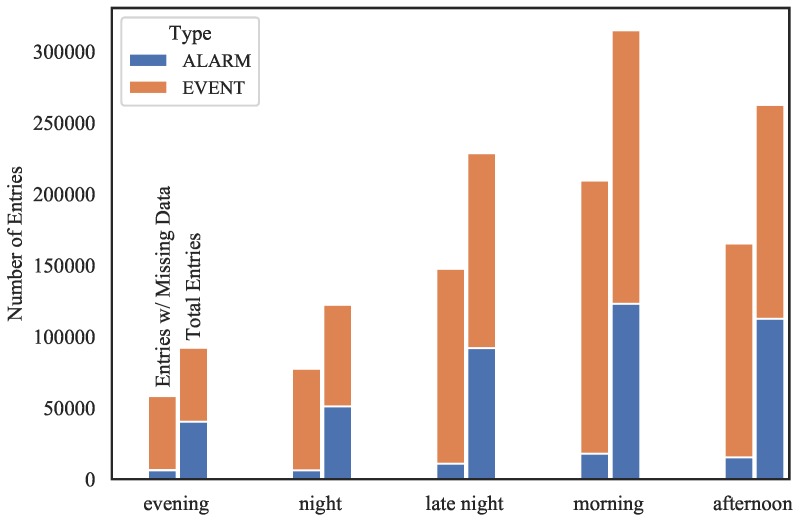
Nullity accounting regarding registry type and time.

Missing data occurrences can be also evaluated by taking the time stamp into account to verify potential relationships among missing data, variables and time. It is possible to see in Figure 6 an overall greater frequency of missing data in event-related records as well as a higher incidence of missing data happening in the morning. This may signalize that configuration or communication issues originating data absences are more prone to industrial events than alarms. Also, that may indicate that processes originating entries with missing data are more likely to occur in the morning, probably due to a higher volume of operations in this period.

When facing a missing data problem, analyzing present data alone as if no data is missing can result in biased results [37]. Still, handling categorical data missingness with imputation requires a cautious strategy as little is known about the missing data mechanism and common imputing approaches in fact yield worse results than simply making a listwise deletion (an entire row is excluded if any single value is missing in that row) [38]. As methods for categorical missing data imputing are rather complex and beyond the scope of an EDA, and since this analysis showed that target data has overall good quality, the exploratory study can go further without strongly requiring a strict missing data handling strategy. However, missing data patterns captured in this step can draw attention of operators and analysts to configuration and communication weaknesses in systems devoted to events and alarm management, which could be addressed to improve data completeness and significance.

### 3.3. Visual Analysis of Categorical Data

The data set under study, as seen in Table 1, has entries basically composed of categorical data which requires a less conventional approach. Qualitative or categorical variables, in distinction from numerical-valued or quantitative variables, assume only a limited number of discrete values and have a category-based measurement scale which can be ordered (ordinal variables) or unordered (nominal variables) [39,40]. Since there is no explicit hierarchy or order among categories of data set variables, all columns are treated in this study as nominal variables. For instance, the variable *Type* differentiates the types of entries in target data and assumes only two categories: *ALARM* and *EVENT*. Clearly, these categories defines if a entry refers to an alarm or an event.

An EDA approach over categorical data may focus on visualization techniques and graphical methods to highlight counts, proportion relationships and patterns between variables and their categories. However, while methods for visualizing quantitative data have a long history, graphical methods for categorical data have only recently developed and, consequently, are not as widely used [41].

#### 3.3.1. Quantitative Assessment

**Figure 7:**
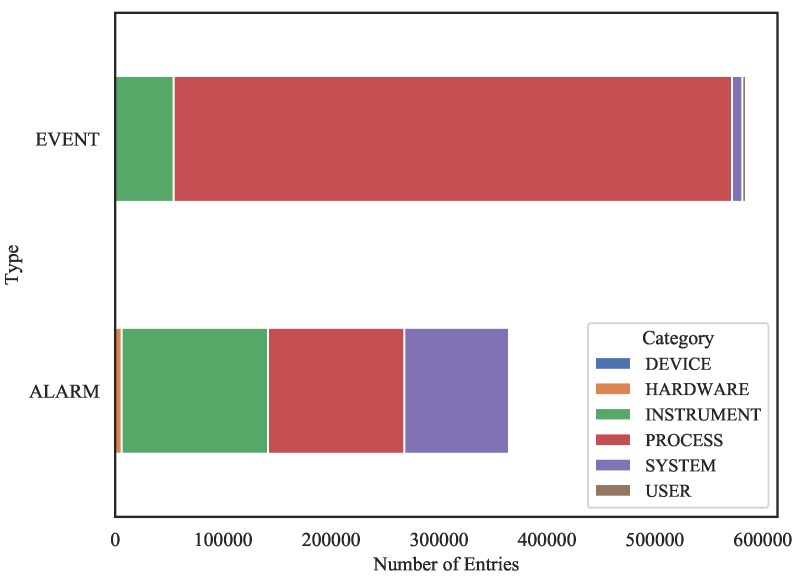
Stacked bar plot associating columns *Type* and *Category*.

**Figure 8:**
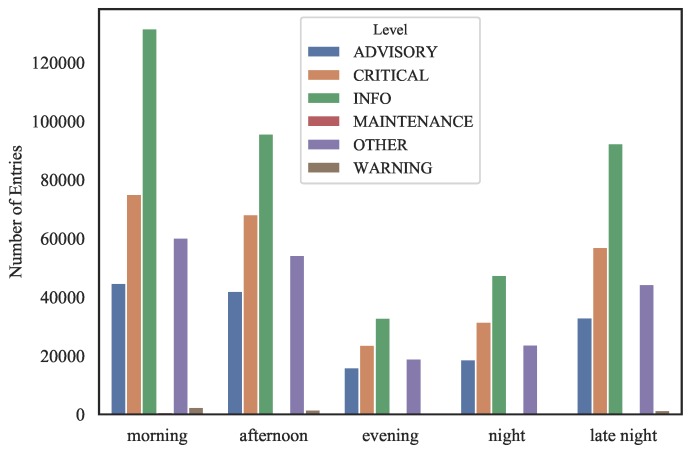
Grouped bar plot accounting *Level* over day-shifts.

A quantitative assessment of categorical occurrences can be easily achieved using a myriad of bar chart types. As example of the various accounts that can be carried out, Figure 7 shows a stacked bar chart evidencing the quantitative relations between columns *Type* and *Category* where it is possible to identify, for example, a high prevalence of a category (in *Category* column) in event entries. Similarly, Figure 8 shows a grouped bar chart accounting column *Level* in relation to the *part_of_the_day* (supplementary categorical variable built from time stamps) where it is possible to visualize the proportions among the several levels (*Level* column) during all times of day. Both bar plots were built using *pandas* [42] and *matplotlib* [43] Python language libraries.

#### 3.3.2. Variables Proportions

Although bar charts are a very straight-forward visualization approach in data analysis, they have limitations when dealing with categorical data. There are some modern visualization methods more suited or specifically designed for categorical data that can provide a better picture of the interrelation among variables proportions and interactions. Among these, mosaic plots [44] and alluvial diagrams [45] are stand outs. These methods are built upon a tabulation of data values that displays the frequencies of each value or group of values in the data set (contingency tables).

**Figure 9:**
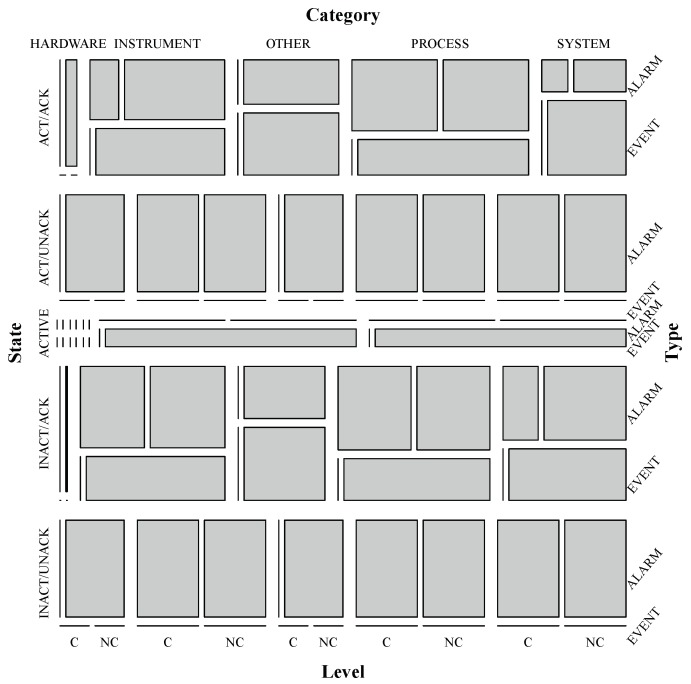
Mosaic plot for columns *Type*, *Category*, *State* and *Level*.

In a mosaic plot, frequencies of data values occurrences in a contingency table are graphically displayed in a collection of rectangular “tiles” whose size (area) is proportional to the cell frequency [41]. Mosaic plots partition plot axes to graphically represent proportions among categorical variables. Although the method can be readily generalized to display n-way tables, due to resolution and overlapping limitations in a graph, using at most 4-way tables (4 variables) is a commonplace. Figure 9 shows a mosaic plot built with R language package *vcd* [46] from a 4-way contingency table regarding variables *Type*, *Category*, *State* and *Level*. In that case, for better visualization, category levels for *Level* variable were grouped in just two levels: *CRITICAL* (C) and *NON-CRITICAL* (NC). This diagram illustrates the big picture for proportional relationships within the categories of those four variables. For instance, it is possible to notice that alarm and events are mostly not critical and the vast majority of *ACTIVE* state entries have *PROCESS* or *SYSTEM* as *Category*.

**Figure 10:**
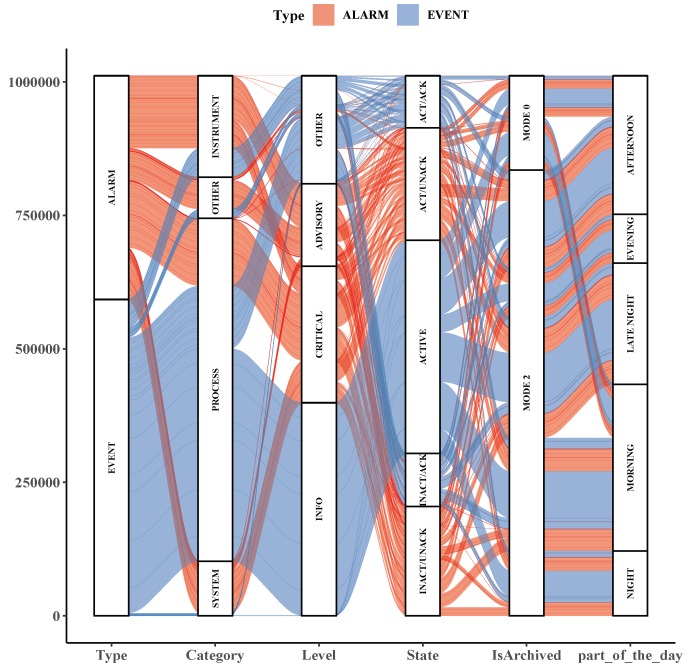
Alluvial diagram for some important columns of the data set.

A more convenient and versatile way of displaying results of categorical variables analysis is the Alluvial diagram. Also built from a contingency table of data set variables values, it allows the visualization of proportional relations among multiple categorical variables at the same time. That can emphasize flow and qualitative dispersion throughout the categories. The R language package *ggalluvial* [47] implements Alluvial diagrams and was used for the most representative columns of the original data set conjoined with the *part_of_the_day* supplementary column as represented in Figure 10. This diagram makes easy to remark the proportional relationship among all variables in the plot. For instance, by seeking the stripes flows, it is possible to attest that *ALARM* entries are very likely to have *INSTRUMENT* as *Category*, *CRITICAL* as *Level* and *ACT/UNACK* as *State*. Still, the majority of *EVENT* entries have *PROCESS* as *Category*, *INFO* as *Level*, *ACTIVE* as *State* and happen between late night and the morning.

**Figure 11:**
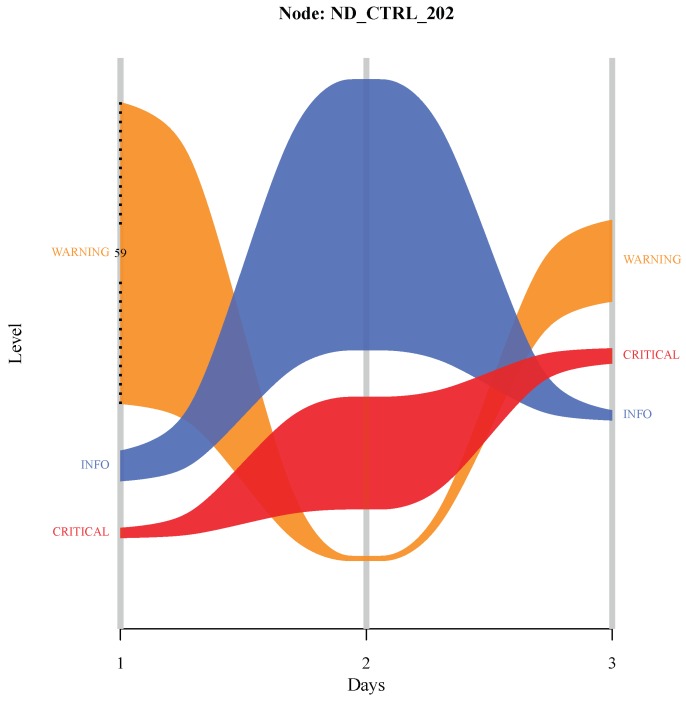
Time series alluvial diagram for a *Node*.

This kind of diagram can also enable a proper visualization of categories frequencies evolution over time. By taking the time component (in days), a target variable (*Level* column) and fixing an individual node (an individual cell in *Node* column), a time series alluvial diagram was built using R language package *alluvial* [48]. Hence, Figure 11 shows *Level* categories frequencies for the node (*Node*) *ND_CTRL_202* over a time period of three days. It is possible to notice interesting occurrences in this time interval. By seeking the flows of the three stripes, it is possible to see a considerable increase in *CRITICAL* entries on day 2, which was preceded by a large increase of *WARNING* and *INFO* entries observed on the previous day. Thence, it is plausible that a series of informational messages and warnings were given on day 1 until critical conditions were reached on day 2 and something close to a normal situation was probably achieved on day 3.

#### 3.3.3. Hierarchy Reasoning

**Figure 12:**
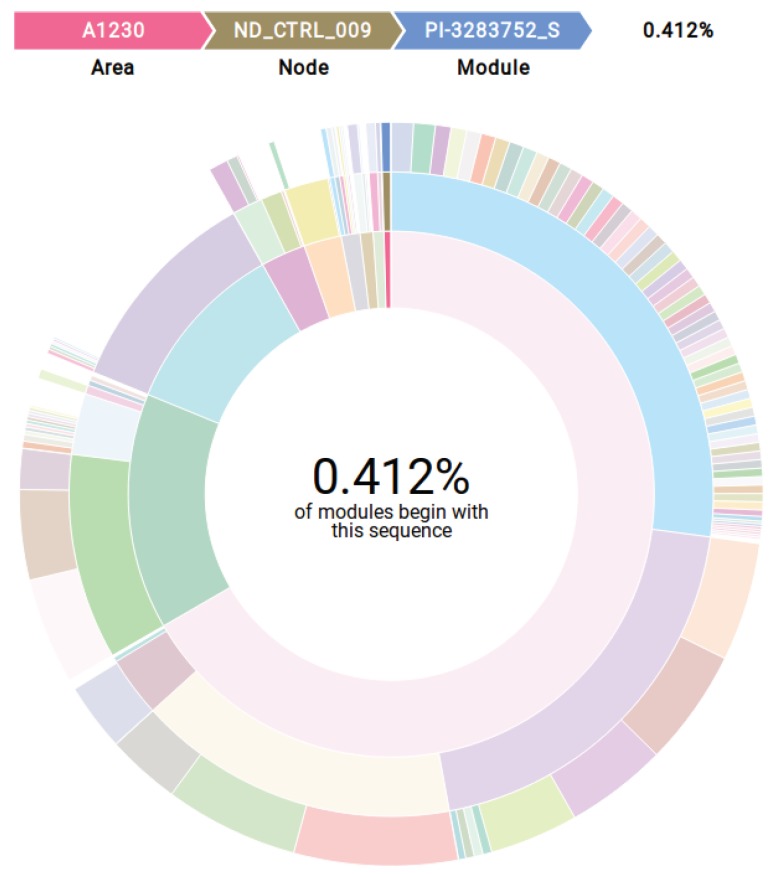
Sunburst diagram highlighting hierarchy for columns *Area*, *Node* and *Module*.

Alarm and event logs refer fundamentally to what is happening to important elements in automation. These elements are arranged in the plant according to a hierarchy that may be reflected in those logs. Visualizing this hierarchy favors a better reconnaissance of the entities that make up the industrial plant. In the case of data under study, a 3-level hierarchy is made explicit by columns *Area*, *Node* and *Module*. This hierarchy can be more easily understood in a quantitative and qualitative manner with the SunBurst diagram, a space-filling visualization technique in which items in a hierarchy are laid out radially, with the top of the hierarchy at the center and deeper levels farther away from the center [49]. To evidence hierarchical relationships and proportional dispositions for the entities covered in the logs, a Sunburst diagram built from that *Area*, *Node* and *Module* columns and implemented with R language package *sunburstR* [50] is shown in Figure 12. This figure makes trivial the understanding of plant hierarchy: each *Area* has several *Nodes*, which in turn can have multiple *Modules*. The breadcrumb trail in top of the figure represents the hierarchy for a selected *Module*.

#### 3.3.4. Mining Descriptions

Some columns in the target data set are descriptive and have high uniqueness for their values. This can make graphical visualizations difficult and hamper analysis. Nevertheless, an overall quantitative and qualitative conjecture is still convenient and some kind of graphical visualization is desirable. This way, an overview of sentence occurrence in a descriptive column can be obtained through word clouds, a popular data mining technique meant to provide a visual emphasis of most frequent words in a text by tidily fitting words in a plane, using font size and color as word frequency differentiators. Word clouds are useful for quickly perceiving the most prominent terms and their relative prominence.

**Figure 13:**
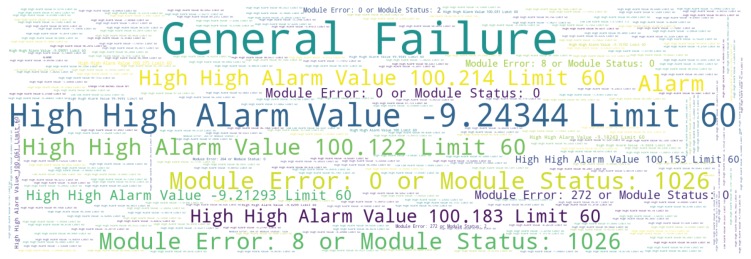
Word cloud from sentences of column *Desc2* for critical alarms.

Using Python language package *wordcloud* [51], a word cloud was built from a frequency table accounting frequencies of sentences in column *Desc2*. This word cloud aims at highlighting the more frequent descriptive sentences, for entries of *Type ALARM* with *Level* set as *CRITICAL*. As this column represents a fine-grained description of entries, by inspecting Figure 13, it is possible to point out the descriptive sentences related to the most common critical alarms in the plant. The word cloud of Figure 13 shows a predominance of “General Failure”, “High High” and “Module Error” alarms, what may indicate that the plant has experienced a troubled period. Although it is not an accurate visualization technique, word clouds are in this case convenient to draw attention to episodes of high frequency and potentially of high significance in the operational context of the plant.

### 3.4. Categories Interactions

An important step in an EDA is to lay emphasis on unveiling possible relationships among variables under analysis. For categorical data, a suited method for this task is Multiple Correspondence Analysis (MCA), an extension of the Correspondence Analysis (CA) for summarizing and visualizing a data table containing more than two categorical variables [52]. MCA is part of Principal Component (PC) methods family whose purpose is summarizing and visualizing the most important information contained in a multivariate data set and can be understood as the categorical data counterpart of Principal Component Analysis (PCA) [52,53].

In short, MCA is data-driven and assumption-free dimensionality reduction method which distributes values of a table of relative frequency or multiple contingencies (Burt table) in an n-dimensional space, and then processes the distance (Chi-square distance) between the variables in each dimension to establish the similarity degree of variables [54,55]. More on MCA method can be comprehensively found in recent specialized books [52,53].

The aim in this step is to proceed MCA to some key variables of the study data set to point out relationships among variables and categories as well as reinforce prior association hypotheses. To perform MCA over target data, *FactoMineR* [56], a R language package dedicated to multivariate EDA focused on classical PC methods, was used. The R language package *factoextra* [57], specialized in the extraction and visualization of PC results, was used for plotting.

**Figure 14:**
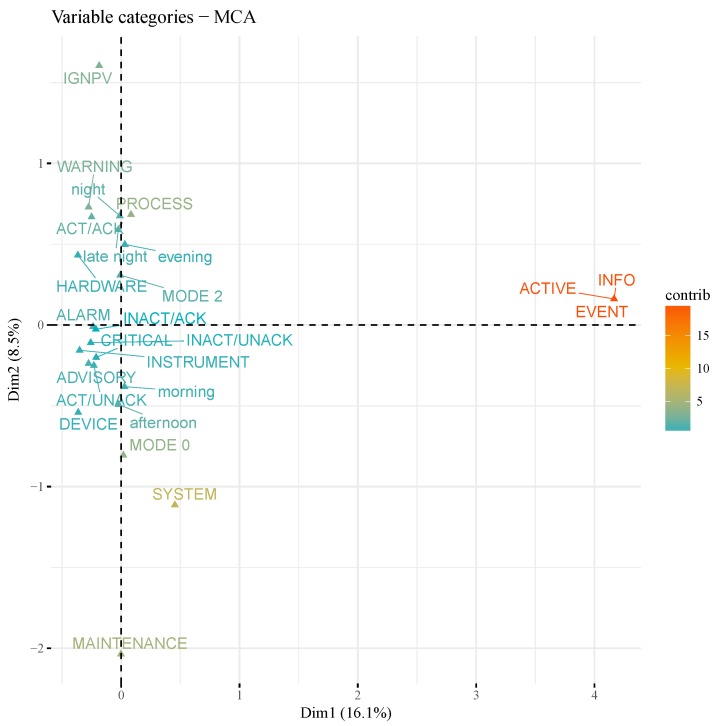
MCA factor map for key variables categories.

As MCA focus primarily on studying the categories, once they represent both variables and a group of individuals [53], variables categories scatterplot (or cloud of categories) was built and represented in Figure 14. In this MCA plot, elements from MCA results are depicted in a so called factor map, a Cartesian plane whose axis are defined by MCA two main dimensions (principal components) and plotted points given by the coordinates of each variable categories in those dimensions. Columns *Type*, *Category*, *State*, *Level*, *IsArchived* and *part_of_the_day* were subjected to MCA and are represented in the scatterplot (Figure 14) by their categories representation in the factor map. Also, axis labels explicit that the two first dimensions retain almost 25% of the total inertia (variation) contained in the data. Categories names are colored according to their contribution values in the factor map, a measure obtained from their squared cosine (cos^2^) which in turn represents the quality of the coordinates in that map.

By interpreting Figure 14, it is possible to figure out that variable categories such as *INFO* (column *Level*), *ACTIVE* (column *State*), *EVENT* (column *Type*), *SYSTEM* (column *Category*), *MAINTENANCE* (column *Level*) and *PROCESS* (column *Category*) contribute the most to the main dimensions. Consequently, they are the most important in explaining the variability in the data set, with *INFO*, *ACTIVE* and *EVENT* being categories more correlated with dimension 1 while *SYSTEM*, *MAINTENANCE* and *PROCESS* are more correlated with dimension 2.

Regarding the relationships between variable categories, much can extracted from the factor map. As an example, it is plausible to say that variable categories with a similar profile are grouped together in the graph. Hence, the well defined cluster formed by categories *EVENT*, *ACTIVE* and *INFO* evinces a stronger relationship (positive correlation) among theses categories when compared to the others. The same way, as negatively correlated variable categories are positioned on opposed quadrants, *WARNING* and *SYSTEM* categories are prone to a mutual exclusion relationship.

## 4. Discussion

This section provides a discussion of the limitations, obstacles and merits regarding this research.

### 4.1. A Superb Guidance But No Definitive Answer

This paper was engaged to contextualize the application of Data Science and Big Data aspects as an undeniable trend in industry. Corroborating with this, the extensive use of EDA, a classic data analysis approach which uses quantitative, qualitative and visual methods, is proposed as a primer step in industrial alarm and event data analysis. EDA is one of several possible approaches in data analysis, but has the advantage of being an uncomplicated “hands-on” approach facilitated by current available tools. In this paper, a basic EDA a pipeline was systematized throughout the gradual understanding of data aspects to obtain from interesting insights to real knowledge relating to data under study. From the examples shown in this work, EDA proved to be able to attain a panoramic view of data, point out general relationships and, consequently, guide further analysis. Although not yielding ultimate conclusions, EDA showed itself effective in extracting valuable information from the kind of data under study. It is worth mentioning that results presented in this work comprises only a small sample of EDA capabilities, used to attest the ability of expressing a hitherto concealed amount of notable importance information, specially to field experts. EDA has a broad scope and can also include deeper statistical analyses which can lead lead to the discovery of cause-effect relationships, serial dependence among registries and more sophisticated patterns.

### 4.2. Use of Real-World Data

The study summarized in this work through a series of graphs and diagrams was conducted using real-world industrial events and alarms data from a petrochemical plant consisting of a small sample related to only a single unit, out of a total of ten, of an industrial process. Selected data refers to a representative scheduling horizon of a typical crude oil processing operation in a petrochemical plant, which lasts for 3 to 4 days [58,59]. This data volume also corresponds to data locally stored in the SCADA system under study and therefore more easily retrievable. The entire plant produces nearly 10 million events and alarm entries a day, totaling about 2 Terabytes of raw data per day of operation, of which almost no advantage is taken. Concerning paper originality, no scientific research on analyzing this kind of industrial data has been identified.

### 4.3. Use of an Open-Source Tool-Set

This research was carried out with the use of an open-source tool-set whose cores comprise Python and R programming languages and their vast ecosystems of 3rd-party libraries related to data science tasks. An open-source research environment was highly preferred over proprietary solutions mainly due to gains in flexibility, scalability and customization obtained with this choice, as well as due to the abundant documentation, fast-growing community of users and and increasing improvement of these tools, allied with a zero-cost setup. This constitutes an inexpensive but powerful environment suited to perform diverse and complex data science tasks. R’s built in Data Frame structure and Python’s *pandas* library, extensively used in this research, are foundation data structuring and manipulation tools upon which all computation tasks can be constructed. With regards to results visualization, R’s *ggplot2* and Python’s *matplotlib* offer a general-purpose and versatile publication-quality image generation environment that was used directly or indirectly through other libraries which rely on them as base graphical systems.

### 4.4. The Need for an Industrial Big Data Infrastructure

This EDA excerpt is part of a research carried out in a offline and controlled laboratory environment, using a conventional computational infrastructure, a set of open-source tools (R and Python libraries) and a small data sample. Perform collection, aggregation, handling and processing of the real data mass of data is indeed a challenge to be overcome. In order to make EDA and other more sophisticated data-driven approaches feasible and viable in such a voluminous data and time-restricted scenario, it is necessary to establish a hardware and software infrastructure capable of meeting the great computational demands of those approaches. Industry has been aligning to some Big Data precepts to accomplish such a kind of processing which, in terms of computational infrastructure, includes, among other aspects, a high computing power, parallel, distributed and online processing of massive data capabilities, distributed file systems with safe access, intelligent, fast and robust communications infrastructure, as well as an analysis systematization based on the specific plant knowledge [12,13,14].

Literature confirms this matter as a high relevance interest topic. A recent contribution concerns the establishment of a Big Data engine able of meeting industrial needs and peculiarities [13]. In that work, the author permeates the potential of a collection of Big Data architectures, optimization approaches and Big Data benchmarking tools in industry and introduces a conception of an industrial Big Data architecture capable of addressing the limitations and improve efficiency of those architectures in relation to their map-reduce models. Another promising reference in this area defines a cloud-assisted architectures and mechanisms for Big Data data processing in the field of preventive maintenance are proposed and compared [15]. Other prominent contribution is a survey focused on key techniques for designing and implementing efficient and high performance industrial Big Data analytics platforms [60]. This recurrence proves that the establishment of an Industrial Big Data infrastructure figures as a requirement for today’s industries.

## 5. Conclusions and Future Work

This work aimed at showing that performing a graphically-rich and informative EDA can pave a shift from where data is simply showed to where a history is told from data. By establishing a low complexity exploratory data analysis pipeline and using adequate visualization methods, valuable information can be obtained from previously neglected (or simply took aside) data. This may lead to improvements in operating, testing, maintenance, monitoring and auditing procedures to ensure safer, more reliable and more effective operations. Apart from that, more appropriate data visualization approaches can also inspire a new-thinking on the design of industrial HMIs, dashboards and panels.

The stages, strategies and methods in this kind of approach are not limited to the ones explored in this research. They must be adapted, extended and deepen according to the nature of the data under analysis, the desired time-window and the goals established for the analysis.

A future work is to enhance and better systematize EDA pipelines in an automated framework able to also consider the domain knowledge supplied by field specialists in order to refine data inquiry and narrow search spaces. Driven by EDA findings, future directions should aim at enriching storytelling by employing more sophisticated analysis strategies such as statistical hypothesis testing, clustering, classification, feature engineering and machine learning to guide diagnostic, prognostic and prediction of abnormal operational situations for different time horizons. A challenge is to make these data analysis improvements in such a voluminous data scenario feasible. Hence, the design, test and build of an industrial Big Data infrastructure is in the roadmap, in a first moment, relying on current leading Big Data solutions that should be set up and configured to meet specific industrial plants criteria.

## Figures and Tables

**Table 1:** Sample entries of data set under analysis.

Date_Time	Type ^a^	Category ^a^	Module	State ^a^	Level ^a^	Desc1 ^b^	...	registry_id ^c^
18-02-21 14:16:04	EVENT	PROCESS	MDL-015783HIC	INACT/ACK	NaN	RATE	...	18794
18-02-23 08:45:36	EVENT	PROCESS	MDL-414572PID	ACTIVE	INFO	ERROR CLEARED	...	12946
18-02-22 02:57:48	EVENT	PROCESS	MDL-155745DI	ACTIVE	INFO	MDL-155745DI/ALM1	...	11846
18-02-23 01:36:37	ALARM	SYSTEM	MDL-689-BVEP	INACT/UNACK	ADVISORY	GENERAL FAILURE	...	13186
18-02-22 12:21:32	ALARM	NaN	MDL-45420VEC	ACT/UNACK	ADVISORY	COMM FAILURE	...	5948
18-02-20 22:18:41	ALARM	SYSTEM	MDL-688-AVEP	INACT/UNACK	CRITICAL	GENERAL FAILURE	...	13361
18-02-23 15:38:42	ALARM	PROCESS	MDL-1557452DI	ACT/UNACK	CRITICAL	HIHI	...	6397
18-02-21 01:55:46	EVENT	PROCESS	MDL-048666TI	ACTIVE	INFO	TI-4283333/AI1	...	23824
18-02-22 16:56:03	ALARM	SYSTEM	MDL-681-BVEP	ACT/UNACK	ADVISORY	GENERAL FAILURE	...	13185
18-02-22 16:41:19	EVENT	INSTRUM	MDL-1029ASMB	INACT/ACK	NaN	MODBAD	...	24883

^a^ Registry attributes; ^b^ Registry description; ^c^ Registry unique identification.
